# Potential of Metallurgical Gradients in the Design of Structural Components Made of Nodular Cast Iron

**DOI:** 10.3390/ma14092411

**Published:** 2021-05-06

**Authors:** Christoph Ripplinger, Markus Gastens, Jannik Zimmermann, Björn Pustal, Christoph Broeckmann, Kai-Uwe Schröder, Andreas Bührig-Polaczek

**Affiliations:** 1 Institute for Materials Applications in Mechanical Engineering IWM—RWTH Aachen University, 52062 Aachen, Germany; c.broeckmann@iwm.rwth-aachen.de; 2 Institute of Structural Mechanics and Lightweight Design SLA—RWTH Aachen University, 52062 Aachen, Germany; jannik.zimmermann@sla.rwth-aachen.de (J.Z.); kai-uwe.schroeder@sla.rwth-aachen.de (K.-U.S.); 3 Foundry Science and Foundry Institute GI, RWTH Aachen University, 52062 Aachen, Germany; b.pustal@gi.rwth-aachen.de (B.P.); office.buehrig-polaczek@gi.rwth-aachen.de (A.B.-P.)

**Keywords:** ductile cast iron, component design, metallurgic gradients, fatigue strength

## Abstract

The objective of this work is to investigate the use of metallurgical gradients (MG) in the design of structural components made of ductile cast iron (DCI). MG have been realized in this study by locally varying the pearlite fraction of the matrix. Exemplarily, the allowable cyclic load for a drive shaft as well as the allowable static displacement are calculated. The performed calculations are based on static and cyclic strength data of four different DCI with amounts of pearlite ranging from 0% to 96.8%. To show the advantage of the purposeful usage of local MG, ten different configurations are examined by numerical simulation studies of a generic drive shaft comprising a circumferential notch. Four configurations are calculated assuming homogenous material throughout the entire component. In the other six configurations the surface region of the notch root has an increased amount of pearlite. For each configuration the allowable multiaxial cyclic load by combinations of torsion and bending was calculated and subsequently the allowable static bending displacement. The results show that the targeted realization of MG results in a significant increase in the multiaxial fatigue strength of the shaft as well as in a slight improvement of the allowable static bending displacement.

## 1. Introduction

Structural components made of ductile cast iron (DCI) are often metallurgically optimized in order to achieve a microstructure as homogeneous as possible. This is due to established guidelines for proof of strength that base on strength parameters published in standards such as DIN EN 1563 [1] considering three categories of microstructures and properties depending on wall thickness. Especially during casting of near net shaped structural components with complex geometries, metallurgical gradients (MG) resulting from heat extraction, nucleation, solidification kinetics and eutectoid transformation are difficult to avoid. Common metallurgical measures usually have side-effects. As a result of these measures a homogenous microstructure is often achieved at the cost of the material properties especially in the highly loaded volumes. In this context a new approach for the design of structural components made of DCI would be to accept microstructural inhomogeneities and, by using the knowhow in controlling the MG, to optimize their internal evolution.

### 1.1. Origin and Control of Metallurgical Gradients in Ductile Cast Iron

The overall quality of DCI is characterized by the amount of ferrite and perlite which is classically adjusted through alloying with Mn and Cu. Other ferrite/perlite stabilizing elements can contribute to the eutectoid transformation such as elevated Si contents in solid-solution strengthened DCI according to DIN EN 1563 [1]. Larger differences in wall thickness in castings and thus varying solidification rates are expressed by a variation in the size, quantity and morphology of the graphite precipitates and the fractions of ferrite and pearlite over several centimeters. The segregations and microstructural inhomogeneities that occur, including both macro- and micro-segregations, can only be controlled to a limited extent by local cooling conditions, such as metallic chills [2,3]. One established procedure to vary the local perlite content is surface heat treatment. Furthermore, the casting surface characteristics can be influenced by means of specifically adjustable contact conditions between melt and molding material [4]. These include locally applied additives [5] as well as the effect of the melt flow and cooling conditions on the solidification kinetics of the surface layer [6]. A lamellar surface layer of approx. 500 µm could be produced by additions of pyrite powder into the coating of a sand core [7]. New combinations of properties (e.g., surface wear-resistant, core ductile) are possible through compound casting, in which melts of different compositions are poured into one cavity. The planning and execution of the processing is usually supported by simulations of the mold filling with regard to the existing cooling conditions [8,9]. The various methods applied for special application show the potential of producing local MG. However, this potential has not been systematically used to design components and to produce and control local gradients during casting.

In the past, different case studies for the purposeful use of MG were performed. Polajnar et al. investigated functionally graded DCI as material for brake pads in the automotive sector [10]. Leibholz et al. investigated the combination of DCI reinforced with tungsten carbide particles [11].

### 1.2. Influence of MG on the Mechanical Properties of DCI

Besides solid-solution strengthening and austempering, the main approach to control the mechanical properties of DCI is through the amounts of pearlite and ferrite in the metallic matrix. By looking at typical DCI grades the strength-enhancing effect of the pearlite for static loads is obvious [12]. For DCI, it is known that the failure originates due to local plastic strains in areas surrounding the graphite nodules [13]. Upon reaching a critical level, these strains result in the formation of interfacial cracks [14]. Increasing loads lead to the circumferential growth of these interfacial cracks until the entire nodule is debonded from the matrix. The resulting voids act as initiation sites for microcracks growing into the matrix perpendicular to the nodules [15]. Applying the same macroscopic global strain to different DCI grades, higher local plastic strains in ferrite surrounding the graphite nodules compared to pearlite were observed [16]. Further results lead to the assumption that the initiation of microcracks in DCI is strain controlled [14]. Thus, it can be explained that the ductile ferrite phase results in the initiation and growth of microcracks at lower global strains or stresses. Comparable to the behavior under static loads, the fatigue strength of DCI is increasing with the pearlite concentration. In [17,18] various DCI grades were characterized in detail. It was shown that increasing tensile strength corresponds to higher cyclic strength, leading to the conclusion that the ratio of ferrite to pearlite also influences the cyclic strength. As an empirical approximation, common strength assessments like [19] allow the estimation of the fatigue strength as a linear function of the ultimate tensile strength (UTS) [20]. These interactions between the pearlite fraction and the mechanical properties result in the idea of utilizing MG locally to enhance components performance.

### 1.3. Calculating Component Strength Considering MG

Conventional design approaches for components made of DCI such as pipes and crankshafts use a constant strength value for the entire component, which is determined experimentally by tensile tests [21]. The test specimen should be representative for the particular casting and is taken either from a separate sample or the appropriate part. ISO 10803 [22] describes the design method for ductile iron pipes against internal pressure and the effects of external loads. Here a nominal ultimate tensile strength value of UTS = 420 MPa is required independent of the wall thickness, whereas the microstructure and, consequently, the local strength is highly influenced by the local wall thickness. In wind power industry DCI is applied in hubs, torque compensators and structural parts in gear boxes such as planet carriers. IEC 61400 [23] describes the design process for a wind turbine gearbox using a constant nominal limit stress, independent of the local geometry or wall thickness. The FKM guideline [19] describes a framework for analytical strength assessment for mechanical components. This guideline also considers only nominal strength values for DCI. In summary, current design guidelines do not take local variations of mechanical strength parameters into account. Therefore, the intentional use of MG in order to control the local strength distribution within the components in the frame of actual design guidelines cannot be used to increase the load bearing capacity of the component. An exception are local heat treatment strategies that induce high strength regions in a component [24]. Nevertheless, such strategies are also not reflected in current general design guidelines.

This study aims to deliver the proof of concept for this innovative approach on component and material design for DCI. In order to achieve this goal, the mechanical strength of DCI with different pearlite contents was determined experimentally. The obtained strength data were then consistently used to predict the maximum load bearing capacity of a generic drive shaft as an example for a typical machine component. This shaft is assumed to be loaded by multiaxial nominal stresses, applied cyclically. Moreover, the shaft has to withstand single extreme loads without catastrophic failure through rupture. This load scenario is typical for components in wind turbine gearboxes. Therefore, a combination of high fatigue strength and fracture strain is required. The proof of the advantages of using metallurgical gradients intentionally is provided by comparing finite element studies, predicting the maximum fatigue strength and bending displacements of metallurgically homogenous and metallurgically graded components.

In the context of this work, metallurgical gradients are defined to be local differences in the microstructure. For the DCI examined here, these differences are represented by locally different amounts of pearlite in the metallic matrix of the component. A systematic use of metallurgical gradients integrated into the component design process, especially in highly stressed local regions, can be used to improve the load bearing of the overall component. This can increase the component safety or, in consequence, make the component lighter by using its lightweight potential. This form of component optimization offers a high potential, which is not yet found in conventional approaches.

## 2. Materials and Methods

### 2.1. Materials

In order to consider a broad spectrum of different pearlite concentrations in the metallic matrix of DCI, four different alloys were examined. Therefore, the ferritic grade EN-GJS-400-18 LT (according to DIN EN 1563 [1], ISO1083/JS/400-18-LT/S according to ISO 1083 [25]), the pearlitic grade EN-GJS-700-2 (ISO 1083/JS/700-2/S) and the ferritic-pearlitic grade EN-GJS-500-7 (ISO 1083/JS/500-7/S) were produced by continuous casting. In order to further modify the ferrite-pearlite ratio the grade EN-GJS-500-7 was also produced by steel mold casting. The chemical composition of each alloy was examined with an optical emission spectrometer (SPECTRO, Kleve, Germany) and is given in Table 1. In order to analyze the microstructure, samples were prepared by grinding, polishing and etching. Nital etching was carried out to make the pearlite visible. The pearlite and ferrite fractions were quantified for multiple micrographs for each cast by the shares of lattice points using linear intercepts of two perpendicular sets of lines (130 × 102 lines). The calculated pearlite fraction results from the ratio of the lattice points on the perlite phase to the total number of lattice points on the metallic matrix. The amount of lattice points on the graphite nodules was eliminated from the total amount beforehand. An optical microscope (Carl Zeiss AG, Oberkochen, Germany) was used. The microstructure of each alloy was evaluated quantitatively in accordance to [26]. Therefore, the parameters’ circularity and compactness, as well as the minimum and maximum feret-diameters were used. The parameters and their calculation are shown in Table 2.

### 2.2. Mechanical Properties

**Figure 1:**
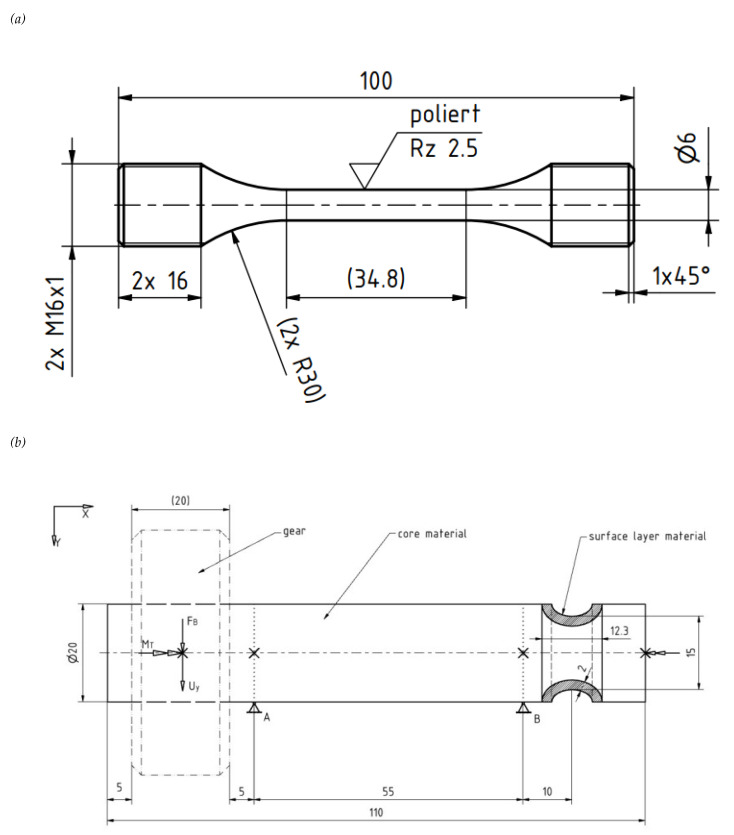
(**a**) Specimen geometry for the fatigue tests (**b**) Geometry and load case of the analyzed generic shaft. All dimensions in mm.

To quantify the static mechanical properties, tensile tests were performed (Zwick Roell ZMart Pro). For each continuous cast, five specimens were tested. In case of the mold cast GJS-500-7 only two specimens were tested. Furthermore, for each continuous cast grade the tension compression fatigue strength σ_W_ (R = −1) was quantified in a fatigue test with at least 15 specimens by the stair step procedure. The ultimate number of load cycles was set to be 10^7^. In case of the mold cast grade, the fatigue strength was estimated from the tensile strength. Therefore, the ratio of σ_W_ over the UTS was calculated for each continuous cast grade and consequently plotted as a function of the pearlite fraction c_P_. (Figure 1a) displays the geometry of the specimens used for the fatigue tests.

### 2.3. Finite Element Model

In order to show the potential of intentionally produced metallurgical gradients in highly loaded areas of a component, an exemplary case study has been carried out on a drive shaft with a flanged gear. This generic part has been chosen, since it is a typical and widespread component, multiaxially loaded and used in many technical applications. By assuming a local MG, expressed as the pearlite fraction in the matrix. A global performance increase of the component is expected. The case study is performed using finite element (FE) modeling. The geometry of the model and the kinematic and static boundary conditions are shown in Figure 1b). The generically selected shaft with a circumferential notch is cyclically loaded by a torsional moment M_T_ and a radial load F_B_. This loading is relevant for the fatigue strength of the component. In the case of single extreme load events the cyclic loading is superimposed by a radial displacement U_y_ applied at the centered load application point and projected on the outer surface in the gear area. This displacement generates a bending load on the shaft. Regarding the design of the shaft, it has to be ensured that it can withstand a sudden failure caused by this extreme bending load. In order to fulfill these particular requirements a ductile core material, allowing for high damage tolerance is advantageous. On the other side, high fatigue strength is required, particular in the notch root area. In this region high strength, which can be obtained in DCI by increasing the pearlite content, would be beneficial. One way to fulfil these requirements, is to introduce a MG, expressed in local pearlite content, into the notch by appropriate alloying and local cooling conditions. In order to take the MG into account in the model, a surface layer is introduced in the notch root. The thickness of this layer is 10% of the shaft diameter. The kinematic boundary conditions are completed by introducing a fixed bearing A and a floating bearing B. At the right shaft face, rotation round the x axis is prevented.

Multiple combinations of core and surface layer material, with changing pearlite fractions, were examined. Four studies were performed, assuming different homogeneous pearlite fractions throughout the entire shaft. Six additional studies were calculated assuming a increased pearlite fractions at the notch.

**Figure 2:**
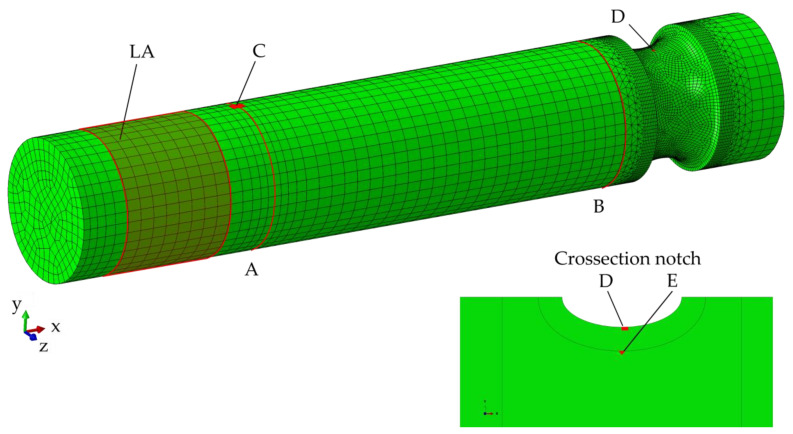
Finite element model with marked load application area (LA), boundary condition (**A**,**B**) and stress extraction locations (**C**–**E**).

**Figure 3:**
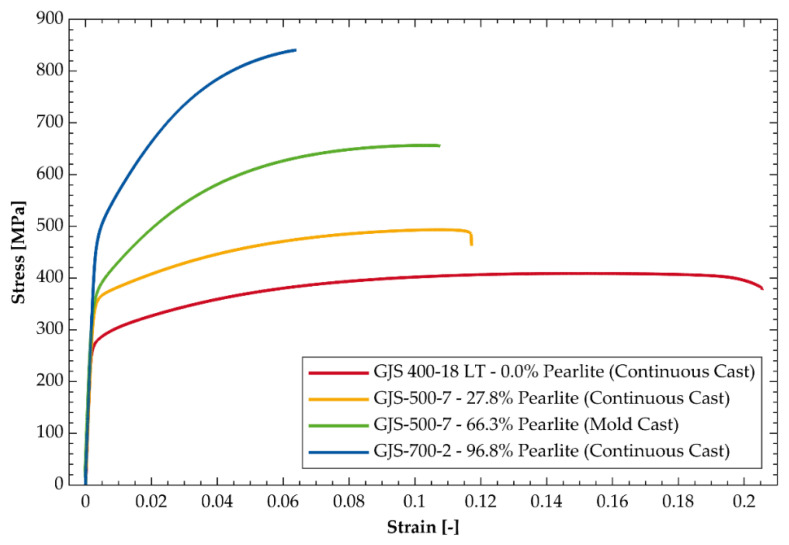
Exemplary stress-strain diagrams for all four materials.

A 3D FEM-model was developed using ABAQUS 2020 (see Figure 2). 8-node linear brick (C3D8R) and 10-node quadratic tetrahedron (C3D10) elements were chosen. The torsional moment is applied at the centered loading point and uniformly transferred to the load application area (LA) of the shaft via a kinematic coupling. The fixed loose bearings (A and B) are each applied to the nodes revolving around the shaft (red edges in Figure 2). The resulting stress data, used for the calculation of the torsional fatigue limit and the tolerable bending displacement are taken from the areas C-E as highlighted in Figure 2. A nonlinear analysis, assuming linear elastic-plastic material behavior (see Figure 3) with piecewise linear hardening in the plastic regime was performed. The used Young’s modulus E, determined by the tangent in the elastic region of the fulfilled tensile tests, and the Poisson’s ratio [27] are given in Table 3. In the first step of the simulation, the torsion superimposed with a radial force F_B_ (proportional to torsion, F_B_/M_T_ = 0.005 × 1/mm) was increased until a degree of utilization of 100% of the fatigue strength according to [19] was reached in the area of the highest torsional stress (D or E, see Figure 2). The applied loads are interpreted as maxima of the amplitudes at R = −1. In the second step the shaft was loaded with 75% of the radial force and torsion calculated in the first step and an increasing static radial displacement U_y_ until the maximum allowable static bending stress according to [19] was reached in the part exposed to the bending load (C), representing a fast extreme bending load case.

## 3. Results

### 3.1. Quantitative Microstructure Analysis

As aforementioned, the microstructure of each sample was analyzed quantitatively concerning the phase composition of the metallic matrix and the morphology of the graphite precipitates. The results are summarized in Table 4.

**Figure 4:**
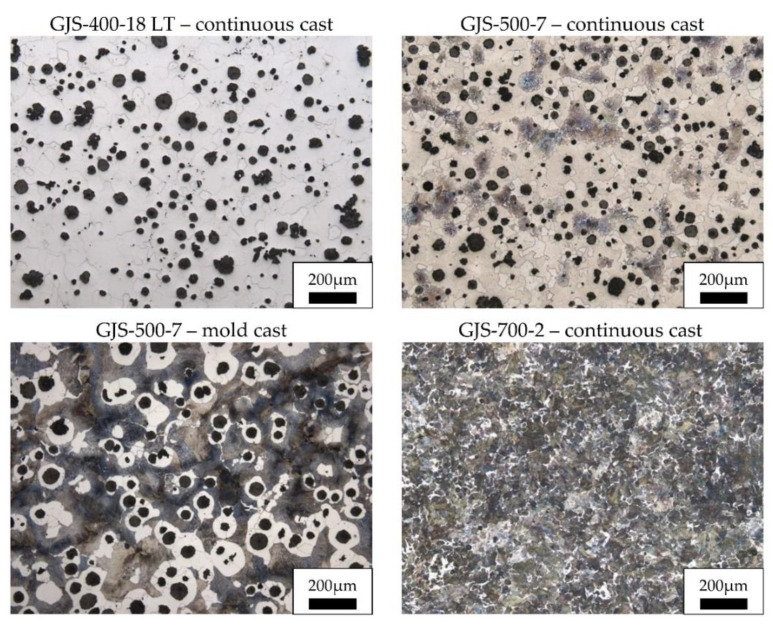
Micrographs of all examined casts, light optical microscopy.

The pearlite fraction of the four cast samples varies between 0% in the ferritic grade GJS-400-18 LT and 96.8% in the pearlitic grade GJS-700-2. For all samples, a comparable size and morphology of the graphite precipitates was observed with the compactness ranging between 90.2% and 94.3%. Exemplary micrographs of all samples after etching with Nital (3% HNO_3_ in isopropyl alcohol; duration depending on sample in the range of 3 to 10s) are shown in Figure 4. It is noticeable, that the pearlite for the continuous cast GJS-500-7 is concentrated in individual closed regions, whereas the matrix of the mold cast variant is mostly pearlitic with ferrite surrounding the graphite nodules. Furthermore, several micropores with sizes corresponded approximately to the graphite nodules were observed for both castings of GJS-500-7. Due to their shape, it is assumed that these pores are not the result of graphite precipitates being extracted during metallurgical preparation.

### 3.2. Tensile Tests

In order to quantify the mechanical behavior of each sample, tensile tests were performed. In Table 3 the resulting average properties as well as the standard deviation for each sample are presented and the stress-strain diagrams are shown in Figure 3. It can be observed that the strength of the samples increases with the fraction of pearlite, while the ductility decreases. The elongation at fracture of the continuous cast GJS-500-7 is lower than expected when compared to the mold cast GJS-500-7 and GJS-400-18 LT.

### 3.3. Fatigue Strength

**Figure 5:**
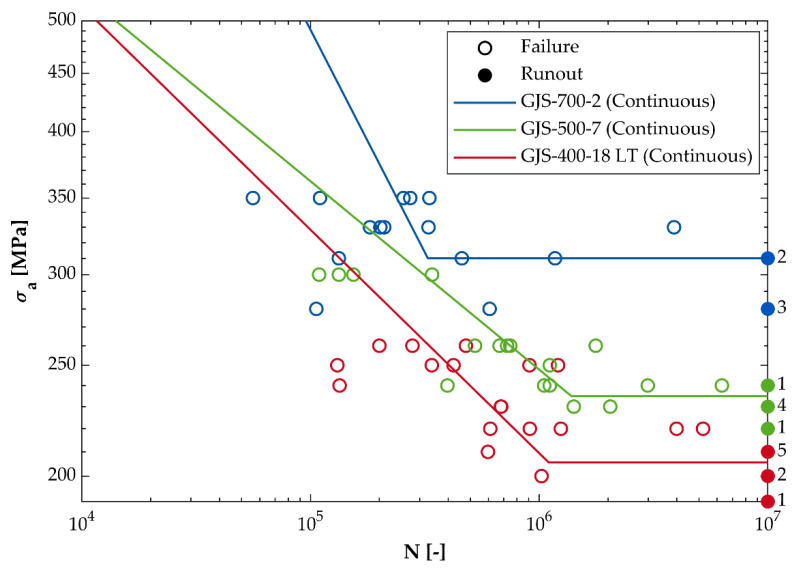
Results of the fatigue tests for the continuous cast grades (straight line: 50% probability of failure).

**Figure 6:**
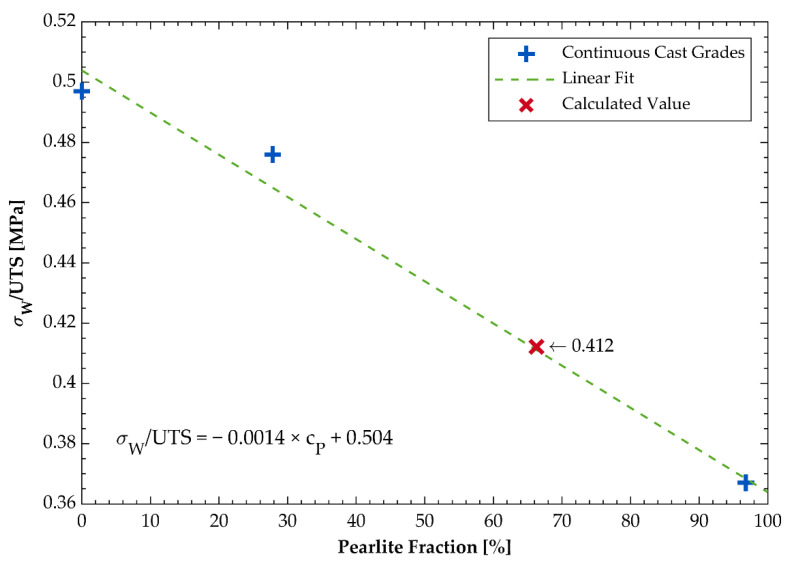
Linear approximation of the ratio of the ratio of the axial fatigue strength over the tensile strength as a function of the pearlite fraction in the matrix.

As aforementioned, the axial fatigue strength (R = −1) σ_W_ of the three continuous cast DCI grades was determined experimentally in a fatigue test. Figure 5 shows the results of these tests. In the case of the mold cast grade GJS-500-7 no fatigue test was performed. To calculate the fatigue strength of this grade, the ratio of the experimentally determined tensile and fatigue strengths of the continuous cast grades was used. The linear interpolation of this ratio as a function of the pearlite concentration was allowable in a good approximation with R² > 98% as shown in Figure 6. This approach resulted in a fatigue strength of σ_W_ = 264.4 MPa for the mold cast GJS-500-7. The ratio between torsional fatigue strength τ_W_ and alternating axial fatigue strength σ_W_ is known as the ductility level T as described in (1).

T = τ_W_/σ_W_ (1)

T is a parameter characterizing the multiaxial fatigue behavior of a particular material and ranges between 0.5 and 1.0. In order to estimate the torsional fatigue strength, in this study T = 0.798 was taken from [28]. Using this value, the torsional fatigue strength of the four DCI grades investigated, was determined according to (1). The fatigue strengths of each sample are listed in Table 5.

### 3.4. Finite Element Model

For this case study, ten FE simulations were performed with different material combinations in the core and surface layer. The shaft configurations with the respective perlite fractions in the core and on the surface can be taken from Table 6. In order to determine the load-carrying capacity of the shaft, the stress data were evaluated in the area of the highest torsional stresses (see Figure 2D or E) and in the area of the highest bending stress (Figure 2C).

### 3.5. Calculation of Allowable Cyclic Torsional Moments and Static Bending Displacements

To compare the different configurations of the shaft, the maximum allowable cyclic torsional moments M_T,max_ and bending forces F_B,max_ as well as the static bending displacements U_y,max_ were calculated. As aforementioned the first step contained the loading of the shaft by a continuously increased torsional moment and bending force which are considered to be the amplitude of a multiaxial alternating load. Consequently, the resulting stress tensors at the positions of the highest stresses (C to E) were used to calculate the cyclic degree of utilization according to [19]. First the cyclic degree of utilization was calculated for the normal stresses A_σ_ and shear stresses A_τ_ separately using (2) and (3). This approach assumes that failure of DCI under normal stresses can be well described by the maximum principal stress. Thus, σ_I_, σ_II_ and σ_III_ denote the principal stresses. Failure due to shear stresses is controlled by the well-known Tresca criteria. Furthermore, a combined degree of utilization A_Comb_ was calculated by using (4) to consider the semi-ductile material behavior of DCI as described in [19]. Thereby A_comb_ is calculated as the sum of A_σ_ and A_τ_ weighted by the empirical material constant q which itself is calculated from the ductility level T with q = 0.264 for any DCI. Finally, for each step of the continuously increased load, the maximum of the three calculated degrees of utilization A_max_ was considered to be critical.

A_σ_ = σ_I_/σ_W_ (2)

A_τ_ = (σ_I_ − σ_III_)/τ_W_ (3)

A_Comb_ = A_σ_ × q + A_τ_ × (1−q) (4)

The allowable cyclic loads for the three positions C to E were determined by linear interpolation between the two increments neighboring A_max_ = 100%. Consequently, the allowable cyclic torsional moment M_T,max_ is the minimum allowable Moment calculated at each of the three positions C to E. The same procedure was used to calculate the allowable bending Force F_B,max_. In Table 6 the values of M_T,max_ and F_B,max_ as well as the critical position are shown for each shaft configuration.

For the subsequent calculation of the maximum allowable extreme load represented by a static bending displacement U_y_ the shaft was initially loaded with 75% of the corresponding M_T,max_ and F_B,max_. These loads are considered to be cyclic. In order to secure a safe approach, the worst-case scenario of the extreme load is assumed to be taking place during the cyclic loads being at their maximum. Therefore, the continuously increasing bending displacement U_y_ was superimposed on the already applied cyclic loads. Similar to the cyclic loads the static degree of utilization A_Stat_ was calculated based on [19] by using Equations (5)–(7).

A_Stat_ = (q × σ_nh_ + (1−q) × σ_eg_)/R_m_ (5)

σ_nh_ = σ_I_ (6)

σ_eg_ = [0.5 × (σ_I_ − σ_II_)^2^ + (σ_II_ − σ_III_)^2^ + (σ_III_ − σ_I_)^2^]^0.5^ (7)

In the Equations (5)–(7) the stress state is described by the principal stresses σ_I_ to σ_III_. The static degree of utilization A_stat_ is calculated as the sum of the maximum-normal stress theory σ_nh_ and the von Mises yield criterion σ_eq_ weighted by the constant q as already described for Equation (4). Since the displacement due to bending represents a single extreme load during the lifetime, for which the catastrophic failure of the shaft due to rupture has to be avoided, a plastic deformation is allowable. Therefore, the maximum allowable bending displacement U_y,max_ corresponds to the largest possible displacement U_y_ which leads to no stresses in the component greater than or equal to UTS. The allowable bending displacement for each position C to E was calculated as the displacement corresponding to A_Stat_ = 100% by linear interpolation between the two neighboring load increments of the FE simulation. The lowest calculated value finally equals to the maximum allowable bending displacement U_y,max_.

**Figure 7:**
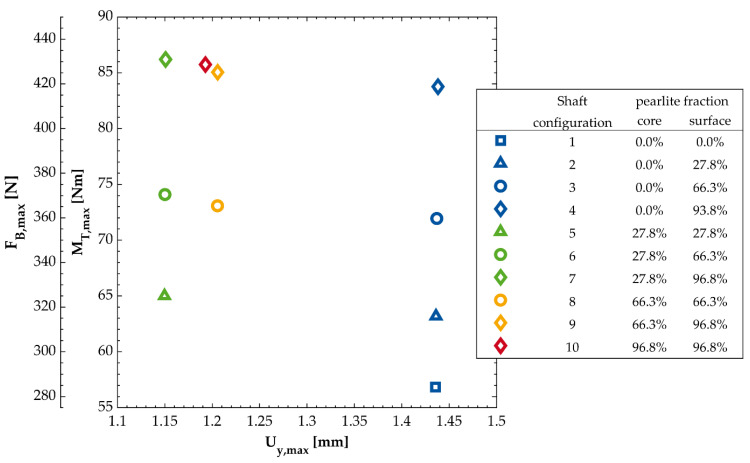
Calculated permissible static bending displacements U_y,max_ as well as cyclic torsional moments M_T,max_ and bending Forces F_B,max_ for each shaft configuration. The individual configurations differ by the concentration of pearlite in the matrix of the core material and the surface layer of the notch.

In Table 7 the calculated U_y,max_ are presented as well as the critical positions of the shaft. Figure 7 contains a graphical representation of the calculated results for M_T,max_, F_B,max_ and U_y,max_ of each shaft configuration.

## 4. Discussion

With regard to the goal of the present work, a significant increase in the fatigue strength of the shaft as a result of the purposeful usage of a pearlite gradient in the notch region is obvious. In case of the configuration with 96.8% perlite in the surface layer the allowable cyclic loads are increased by 47.3% compared to the shaft configuration 1 assuming a homogeneous ferritic matrix. Furthermore, the fatigue strength of the configuration 4 is only 2.3% below the purely pearlitic shaft. Thereby the design of components with an increased fatigue strength in the highly loaded volume and a good ductility in the remaining volume is possible.

Looking at the configurations 5 to 7, the low values for U_y,max_ is noteworthy. This is most probably a result from the comparably low elongation at fracture of the continuous cast grade GJS-500-7 consequently resulting in low allowable strains during the static bending displacement.

In addition to the increase of the fatigue strength for each shaft configuration with an increased pearlite content in the surface layer a slightly increased allowable displacement U_y,max_ of up to 0.2% was observed. No graded shaft configuration showed a decrease in M_T,max_, F_B,max_ or U_y,max_ compared to the homogenous shaft with the same pearlite concentration in the core.

The description of the shaft with a sharp change in the pearlite concentration between the core and the surface is a significant simplification, as a result of which the calculated results for the graded shafts are not to be taken as comparative values for practical applications. Furthermore, the underlying values for τ_w_ were calculated using an empirical approach. Nevertheless, the established tendencies reliably prove that the purposeful usage of MG for DCI can significantly improve the performance of components. This performance increase can be used to design safe light weight components by locally increasing the ductility and fatigue strength.

Today the usage of gradients is often restricted to so called functionally graded materials which rely on complex manufacturing processes like hybrid casting. The underlying approach of this work is the targeted adjustment of MG during the casting of components. Thereby it is suitable for a wide variety of components and promises a cost-efficient way of using MG to enhance performance. Further research on this topic is needed to allow for a safe prediction of the local mechanical properties resulting from the casting process parameters.

## 5. Conclusions

The goal of this work—to prove the advantage of metallurgical gradients in component design—was achieved for the use case of a generic drive shaft made of ductile cast iron. By increasing the pearlite concentration and thereby the static and fatigue strength in the near-surface region at a notch, a significant increase of the fatigue strength was achieved. No influence of an increased pearlite fraction in the surface area on the permissible bending displacement due to an extreme load was observed.

## Figures and Tables

**Table 1:** Chemical composition of the examined alloys of DCI in weight percent.

DCI Grade	Sample	Fe	C	Si	Mn	P	S	Cr	Mo	Ni	Al	Co	Cu
GJS-400-18 LT	Continuous	92.9	3.71	2.47	0.12	0.015	0.010	0.016	0.006	0.30	0.0126	0.01	0.013
GJS-500-7	Continuous	92.4	2.93	2.72	0.62	0.017	0.012	0.043	0.008	0.04	0.0102	0.01	0.988
GJS-500-7	Mold	92.9	3.90	2.30	0.21	0.014	0.015	0.025	0.005	0.07	0.0089	0.01	0.344
GJS-700-2	Continuous	92.1	3.24	2.71	0.62	0.016	0.011	0.044	0.008	0.04	0.0106	0.01	0.990

**Table 2:** Parameters for the quantitative description of graphite morphology.

Maximum feret diameter	Maximum diameter of a graphite nodule	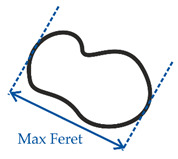
Minimum feret diameter	Minimum diameter of a graphite nodule	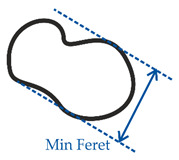
Circularity	=(Nodule area/(0.25 × pi × MaxFeret²))^0.5	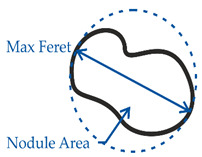
Compactness	=Nodule Area/convex hull area	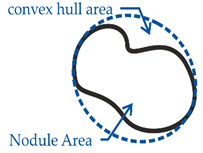

**Table 3:** Results of the tensile tests for each sample.

DCI Grade	Sample	Young’s Modulus [GPa]	Poisson’s Ratio [27] [-]	Perlite Concentration [-]	Yield Strength [MPa]	Tensile Strength (UTS) [MPa]	Uniform Elongation [%]
GJS-400-18 LT	Continuous	151.4	0.28	0.0%	281.9 ± 1.4	414.0 ± 3.2	17.55 ± 0.28
GJS-500-7	Continuous	169.3	0.28	27.8%	360.2 ± 2.7	494.0 ± 3.9	13.53 ± 1.14
GJS-500-7	Mold	160.9	0.28	66.3%	373.8 ± 9.0	641.0 ± 22.0	11.40 ± 0.34
GJS-700-2	Continuous	165.9	0.28	96.8%	514.5 ± 13.2	844.6 ± 9.4	6.00 ± 1.13

**Table 4:** Microstructure of the examined DCI casts.

DCI Grade	Sample	Perlite Fraction [-]	Circularity [-]	Compactness [-]	Max Feret [µm]	Min Feret [µm]
GJS-400-18 LT	Continuous	0.0%	67.0%	91.4%	26.4	20.2
GJS-500-7	Continuous	27.8%	70.1%	90.2%	38.8	30.8
GJS-500-7	Mold	66.3%	71.2%	93.7%	26.7	21.2
GJS-700-2	Continuous	96.8%	72.4%	94.3%	25.9	20.7

**Table 5:** Axial and torsional fatigue strength of each sample.

DCI Grade	Sample	Perlite Fraction [-]	σ_W_ [MPa]	τ_W_ [MPa]
GJS-400-18 LT	Continuous	0.0%	205.6	164.0
GJS-500-7	Continuous	27.8%	235.0	187.5
GJS-500-7	Mold	66.3%	264.2	210.8
GJS-700-2	Continuous	96.8%	310.0	247.3

**Table 6:** Maximum allowable cyclic load M_T,max_ and F_B,max_.

Shaft Configuration	Pearlite Fraction		M_T,max_ [Nm]	F_B,max_ [N]	Critical Position
	Core [-]	Surface [-]			
1	0.0%	0.0%	56.86	284.28	D
2	0.0%	27.8%	63.19	315.94	D
3	0.0%	66.3%	71.94	359.70	D
4	0.0%	93.8%	83.75	486.84	D
5	27.8%	27.8%	65.00	325.01	D
6	27.8%	66.3%	74.09	370.44	D
7	27.8%	96.8%	86.19	511.68	D
8	66.3%	66.3%	73.08	365.40	D
9	66.3%	96.8%	85.04	425.22	D
10	96.8%	96.8%	85.73	428.67	D

**Table 7:** Maximum allowable static bending displacement U_y,max._

Shaft Configuration	Pearlite Fraction		U_y,max_ [mm]	Critical Position
	Core [-]	Surface [-]		
1	0.0%	0.0%	1.4355	C
2	0.0%	27.8%	1.4361	C
3	0.0%	66.3%	1.4369	C
4	0.0%	93.8%	1.4383	C
5	27.8%	27.8%	1.1496	C
6	27.8%	66.3%	1.1500	C
7	27.8%	96.8%	1.1507	C
8	66.3%	66.3%	1.2055	C
9	66.3%	96.8%	1.2058	C
10	96.8%	96.8%	1.1928	C

## Data Availability

The data presented in this study are available on request from the corresponding authors.
